# HSF1 Is Essential for the Resistance of Zebrafish Eye and Brain Tissues to Hypoxia/Reperfusion Injury

**DOI:** 10.1371/journal.pone.0022268

**Published:** 2011-07-21

**Authors:** Nathan R. Tucker, Ryan C. Middleton, Quynh P. Le, Eric A. Shelden

**Affiliations:** 1 School of Molecular Biosciences, Washington State University, Pullman, Washington, United States of America; 2 Center for Reproductive Biology, Washington State University, Pullman, Washington, United States of America; Charité Universitaetsmedizin Berlin, Germany

## Abstract

Ischemia and subsequent reperfusion (IR) produces injury to brain, eye and other tissues, contributing to the progression of important clinical pathologies. The response of cells to IR involves activation of several signaling pathways including those activating hypoxia and heat shock responsive transcription factors. However, specific roles of these responses in limiting cell damage and preventing cell death after IR have not been fully elucidated. Here, we have examined the role of heat shock factor 1 (HSF1) in the response of zebrafish embryos to hypoxia and subsequent return to normoxic conditions (HR) as a model for IR. Heat shock preconditioning elevated heat shock protein expression and protected zebrafish embryo eye and brain tissues against HR-induced apoptosis. These effects were inhibited by translational suppression of HSF1 expression. Reduced expression of HSF1 also increased cell death in brain and eye tissues of embryos subjected to hypoxia and reperfusion without prior heat shock. Surprisingly, reduced expression of HSF1 had only a modest effect on hypoxia-induced expression of Hsp70 and no effect on hypoxia-induced expression of Hsp27. These results establish the zebrafish embryo as a model for the study of ischemic injury in the brain and eye and reveal a critical role for HSF1 in the response of these tissues to HR. Our results also uncouple the role of HSF1 expression from that of Hsp27, a well characterized heat shock protein considered essential for cell survival after hypoxia. Alternative roles for HSF1 are considered.

## Introduction

A low tissue oxygen concentration, followed by the reintroduction of oxygen, gives rise to a phenomenon known as ischemia/reperfusion injury, and is central to the progression of numerous human pathologies [1]. Current treatments are essentially limited to restoring blood flow to the affected region and therapy to diminish negative effects of the injury. Although cells have mechanisms to ameliorate the effects of resulting damage, the harmful intracellular conditions accompanying IR often override these defenses, causing necrotic or apoptotic cell death. This has particularly devastating consequences for tissues of limited regenerative ability, such as the brain and retina [2], [3]. Numerous studies have characterized mechanisms underlying the resistance of cells to IR injury (for representative review, see [4]), however, the potential contributions of various specific proteins and pathways to overall cell survival remains unclear. Thus, further understanding of protective pathways and cellular responses to ischemia/reperfusion injury may lead to development of improved or novel clinical therapies.

Heat shock proteins (HSPs) are produced by cells in response to stress and play central roles in regulating cell survival, as well as protein folding and degradation [5]. HSPs are expressed by cells in response to a wide variety of adverse stimuli including hypoxia, hyperthermia, oxidative stress, UV light, radiation, and exposure to metal toxins. Importantly, HSP expression driven by exposure to one stressor can protect cells against a subsequent stress, which may be of a different type, a phenomenon termed “preconditioning”(see [6] for a representative review). For example, transient hyperthermia upregulates HSP expression in cells and intact tissues and can afford protection against subsequent ischemic injury [7], [8], [9]. Stress-inducible HSP expression is often controlled by activation of heat shock inducible transcription factors, primarily HSF1 [10]. Activation of HSF1 has been demonstrated in kidney [11], [12], heart [13], and brain [14] after ischemia, as well as cultured cells subjected to chemical ATP depletion [12]. In addition, introduction of an HSF1 decoy promoter into renal tubular epithelial cells exacerbates cellular injury after anoxia [15], and hearts of transgenic mice expressing constitutively active HSF1 are more resistant to ischemia than those of wild-type mice [8]. In the rat brain, treatment with geldanamycin to activate HSF1 reduced infarct size and apoptotic cell death after cerebral artery occlusion [16]. However, to our knowledge, loss of function studies examining the role of HSF1 in intact ischemic brain or eye have not been conducted.

The present study addresses the function of HSF1 in brain and eye tissues of the zebrafish embryo using gene knockdown approaches. To simulate IR, embryos were immersed in hypoxic medium for varying times followed by return to oxygenated medium, which we term hypoxia/reperfusion (HR). Although studies examining the role of HSF1 in specific tissues exposed to HR have not been previously conducted using this model, zebrafish embryos are sensitive to hypoxia [17] and express a homologue of mammalian HSF1 [18]. In addition, morpholino knockdown of HSF1 results in enhanced sensitivity of zebrafish embryos to heat shock [19]. Results of the present study demonstrate that heat shock preconditioning elevates Hsp27 and Hsp70 expression in zebrafish embryos and reduces both embryo mortality and apoptotic cell death in brain and eye of zebrafish embryos after HR. In addition, our results demonstrate that HSF1 is required for preconditioning dependent and independent resistance to HR. However, although heat shock protein expression was induced by HR alone as well as by heat shock preconditioning, HSF1 knockdown resulted in only a moderate decrease in inducible Hsp70 expression and no detectable reduction in Hsp27 expression in embryos subject to HR without heat shock preconditioning. These results establish the zebrafish embryo as a model for the study of ischemic injury in brain and eye tissues and suggest an unconventional role for HSF1 in cellular resistance to ischemia/reperfusion injury.

## Results

### Effects of HR on survival of zebrafish embryos

**Figure 1:**
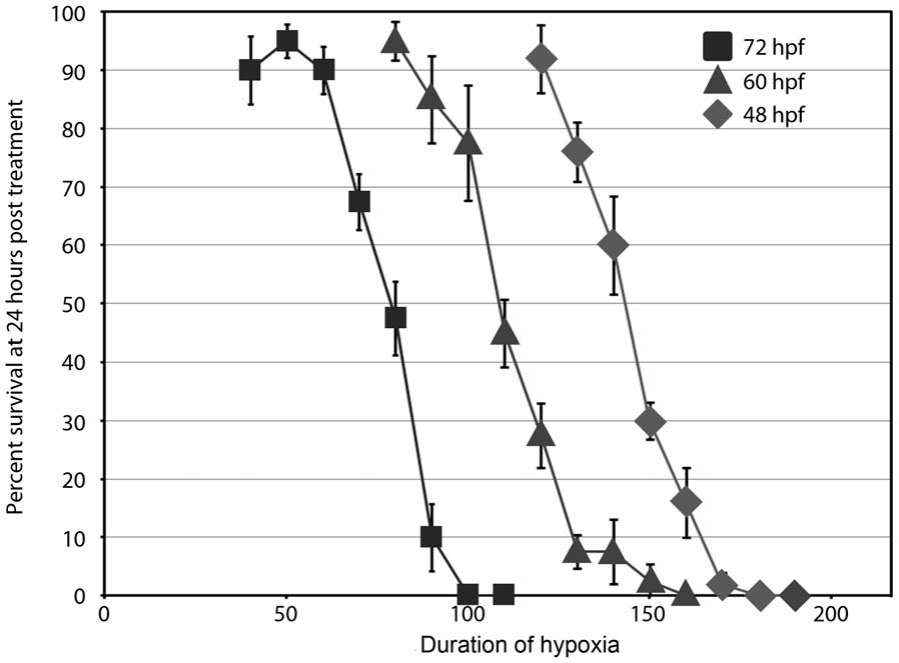
Effects of hypoxia on survival of zebrafish embryos. Zebrafish embryo survival was examined after exposing 48 (diamond), 60 (triangle), and 72 (square) hour-old embryos to hypoxic conditions for the indicated times. Survival was evaluated after 24 hours of recovery in normoxic medium. Data points represent the average percentage of embryo survival in 5 trials. Error bars are the SEM.

Previous studies have characterized the survival of zebrafish embryos subjected to low oxygen conditions up to 48 hours post fertilization (hpf) [17]. As significant development occurs after that time, we examined the response of older embryos to HR. 48, 60, and 72 hpf embryos were incubated in fish water containing low oxygen (0.08–0.12 ppm O_2_) and survival, defined as the absence of opaque tissues and the presence of a beating heart, was assessed 24 hours post treatment. Resulting survival curves are shown in Figure 1. LD50s are approximately 145 min for 48 hpf embryos, 110 min for 60 hpf embryos, and 80 min for 72 hpf embryos, indicating an increasing susceptibility to HR with increasing age.

### Heat shock preconditioning elevates heat shock protein expression and protects zebrafish embryos against HR injury

**Figure 2:**
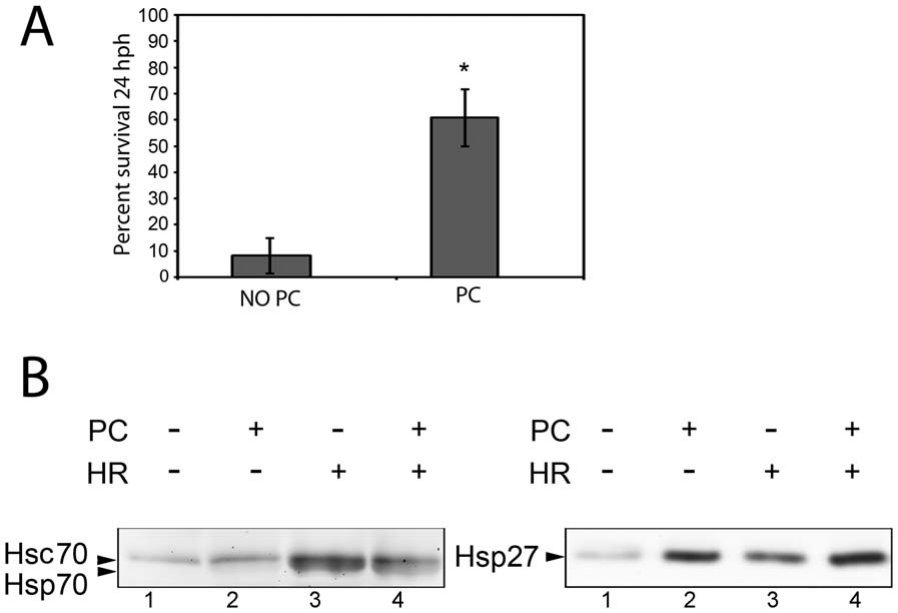
Effect of heat shock preconditioning on embryo survival and heat shock protein expression. A: Average percentage survival of embryos treated at 48 hpf with 160 min of hypoxia. Embryos were either heat shock preconditioned (PC) or not (No PC) and analyzed for survival 24 hours post hypoxia. Data shown are from five independent experiments. Error bars are the SEM. The asterisk indicates a statistically significant difference. B: Representative Western blots showing Hsp70 and Hsp27 expression after heat shock preconditioning and/or hypoxia treatments.

Heat shock preconditioning is considered to be a hallmark of HSF1-dependent cytoprotection (see [20], for example). However, the ability of heat shock preconditioning to protect zebrafish embryos against HR injury has not been previously established. To address this, 34 hpf embryos were preconditioned at 37°C for 90 min and allowed to recover at 28°C for 14 hours. Embryos were then subjected to 160 min of hypoxia at 28.5°C and returned to normoxic conditions for another 24 hours. Over five independent experiments, average survival of non-preconditioned (NO PC) embryos was 8.3%, whereas average survival of preconditioned (PC) embryos was 61.0% (Fig. 2A). Therefore, heat shock preconditioning produced a dramatic and statistically significant (p<.001) increase in survival of embryos subjected to subsequent HR.

**Figure 3:**
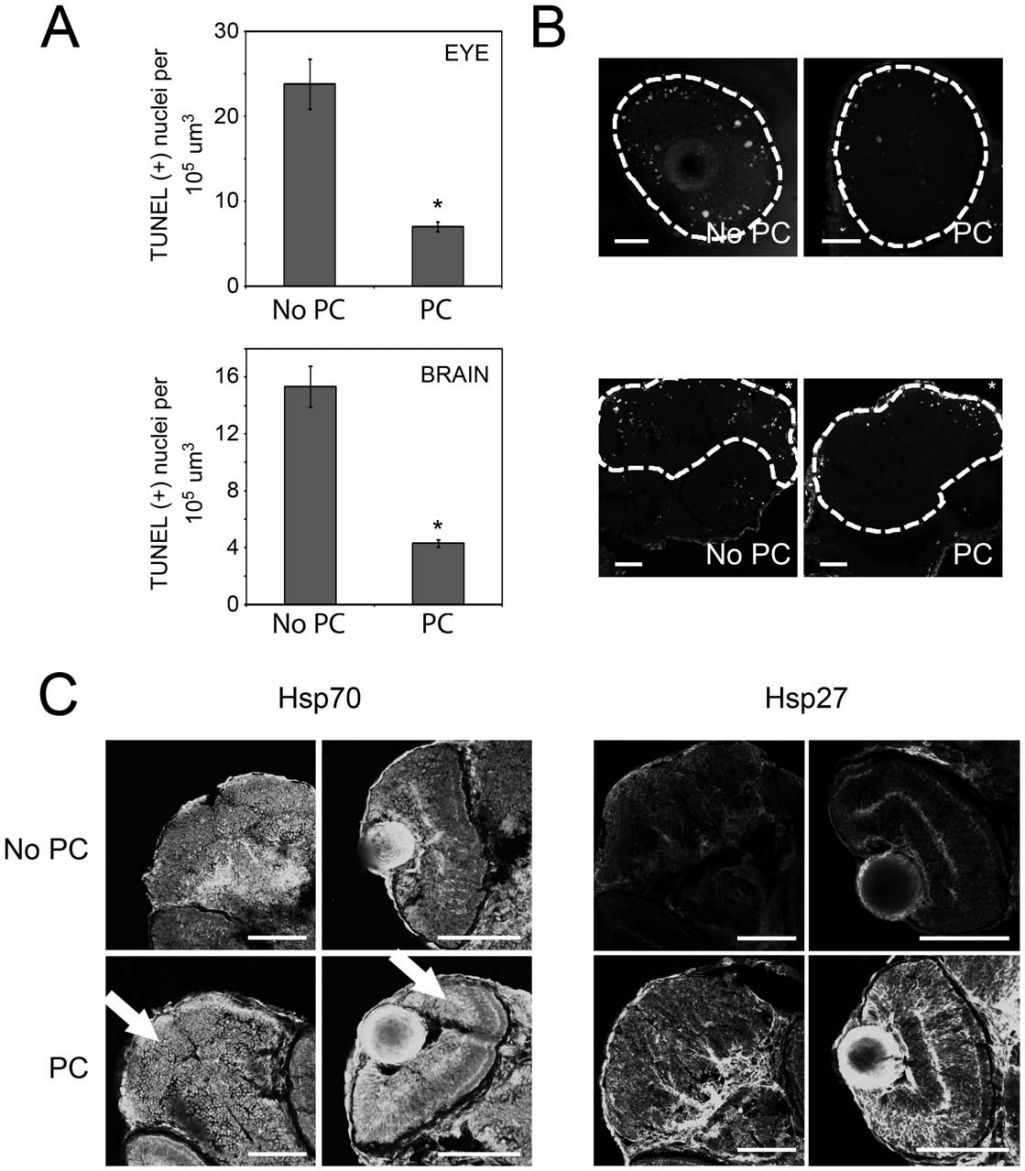
Effects of heat shock preconditioning on cell death in zebrafish brain and eye tissues after sublethal hypoxia treatments. A: The average number of TUNEL-positive nuclei per unit volume in the eye and brain following 100 min of hypoxia at 48 hpf and 8 hours of recovery in normoxic media. Asterisks indicate statistically significant differences between values obtained from non-preconditioned (No PC) embryos and embryos preconditioned with a heat shock at 34 hpf (PC). Error bars are the SEM. B: TUNEL positive cells in representative images of eye (upper panels) and brain (lower panels) regions after hypoxia. Dashed lines indicate the region in which nuclei were counted. Scale bars are 100 µm. Asterisks mark the dorsal, anterior aspect of the embryos. C: Hsp70 and Hsp27 immunostaining of brain (left panels) and eye (right panels) tissues. Arrows highlight regions showing increased Hsp70 staining intensity of heat shock preconditioned (PC) tissues compared to non-preconditioned (No-PC) controls. Increases in Hsp27 staining intensity are also evident throughout the preconditioned tissues. Scale bars are 100 µm.

Next, we assayed expression of representative members of the high and low molecular weight HSPs Hsp70 and Hsp27 in embryos subjected to heat shock preconditioning (PC), hypoxia/reperfusion injury (HR), or both. These proteins have been previously implicated in protection of cells against ischemic injury (for reviews, see [21], [22], [23]). Because 160 min of hypoxia was lethal to a majority of 48 hpf embryos (Fig. 1), embryos in this study were exposed to hypoxic conditions at 48 hpf for only 100 min. This treatment was sublethal for 100% of the embryos (data not shown). Heat shock protein expression was evaluated at 58 hpf. Heat shock preconditioning (PC) resulted in an increase in Hsp70 and Hsp27 expression in embyros (Fig. 2B, compare lanes 1,2). Hypoxia/reperfusion injury also resulted in increased expression of these proteins (Fig. 3, compare lanes 1,3). Finally, combined heat shock preconditioning and subsequent HR also elevated Hsp70 and Hsp27 expression relative to untreated controls (Fig. 2, compare lanes 1,4). However, it does not appear that the combination of stresses produced an additional increase in Hsp70 expression, relative to that produced by individual stresses. In contrast, treatment of embryos with both stressors (Figure 2B, compare lanes 2,3 and 4) leads to higher expression of Hsp27 than individual stressors. Interestingly, our results suggest that Hsp27 expression is increased in zebrafish embryos exposed to heat shock preconditioning to a greater extent than that of Hsp70, while Hsp70 expression appears to be more responsive to HR. The results shown are representative of three independent trials and blots.

The above studies (Fig. 2A) demonstrate that zebrafish embryos display a heat shock response that is protective against subsequent lethal HR. However, clinically relevant brain and eye impairment in humans occurs in response to local, sublethal ischemia/reperfusion injury. To examine the role of the heat shock response in zebrafish embryos exposed to this type of injury, we compared apoptotic cell death in brain and eye tissues of embryos exposed to sublethal (100 min) hypoxia with and without heat shock preconditioning (Fig. 3). Quantitative analysis of the average number of apoptotic nuclei per unit volume (100,000 µm^3^) is shown in Figure 3A. The accompanying images (Fig. 3B) are representative projections of confocal z-series with the number of apoptotic nuclei closest to the corresponding averages shown in Figure 3A. Apoptosis in the brains of 58 hpf untreated controls was negligible, and heat shock preconditioning alone did not increase this value (data not shown). However, 100 min of hypoxia in the absence of heat shock preconditioning (NO PC) at 48 hpf increased the number of apoptotic nuclei detected at 58 hpf to an average of 23.8+/−2.9 nuclei per unit volume in the eye and 15.3+/−1.4 nuclei in the brain. In contrast, the number of apoptotic nuclei per unit volume in heat shock preconditioned embryos was 12.5+/−0.6 (eye) and 11.0+/−0.3 (brain). These latter values were statistically different from, and approximately 70% less than, values obtained from tissues of embryos subject to HR without preconditioning. To confirm that heat shock protein expression in brain and eye tissues were elevated by heat shock preconditioning, we conducted immunolocalization of Hsp70 and Hsp27 in these tissues (Fig. 3C). For both Hsp70 and Hsp27, fluorescent signal was increased in eye and brain following heat shock preconditioning. Additionally, similar to results shown in Figure 2B, preconditioning increased the expression of Hsp27 to a greater extent than that of Hsp70.

### HSF1 expression is reduced in zebrafish embryos by morpholino oligonucleotide microinjection

**Figure 4:**
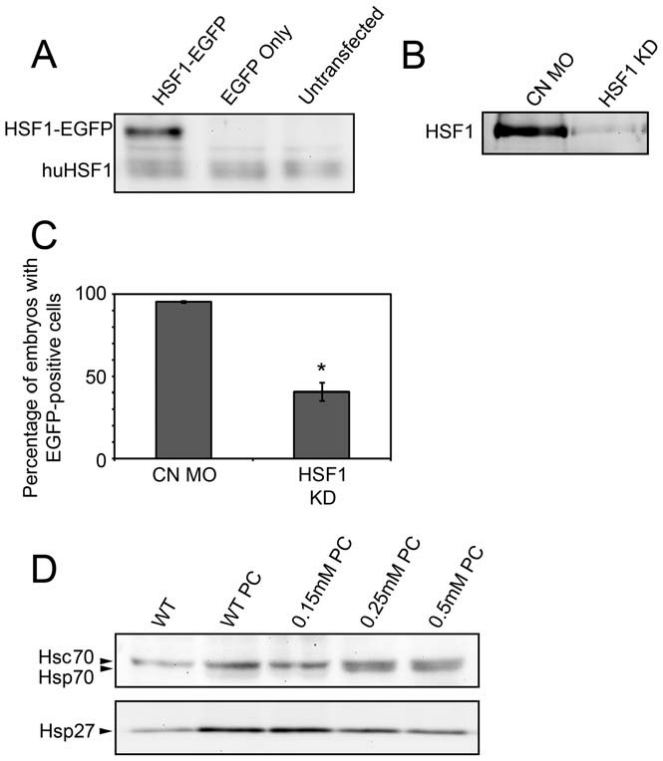
Assessment of HSF1 knockdown following HSF1 MO injection. A: Western blots of HeLa cell protein using the SPA-901 anti-Human HSF1 antibody. A single band is seen at the predicted molecular weight of human HSF1 (huHSF1) in all lanes. An additional higher molecular weight band is detected in extracts of cells transfected with plasmid coding for a zebrafish HSF1-EGFP fusion protein (left lane), but not EGFP alone (middle lane). B: Western blot of HSF1 in nuclear extracts of 48 hpf zebrafish embryos. Embryos were injected with control (CN MO) or anti-HSF1 (HSF1 KD) MO. C: Quantification of the number of embryos containing fluorescent cells following co-injection of a HSF1-EGFP reporter plasmid and control (CN MO) or HSF1 antisense (HSF1 KD) MO. Error bars are the SEM. The asterisk indicates a statistically significant difference between α-HSF1 and CN-MO injected embryos. D: Effect of α-HSF1 MO on heat shock protein expression after heat shock preconditioning (PC). Embryos were injected with the indicated concentrations of α-HSF1 MO or uninjected (WT), followed by Western blotting for the detection of Hsp70 and Hsp27.

To investigate the role of HSF1 in zebrafish embryos after HR, we conducted loss of function studies by injecting zebrafish embryos with a previously described antisense HSF1 morpholino oligonucleotide (α-HSF1 MO) designed to specifically inhibit translation of zebrafish HSF1 mRNA [19], [24]. The previous authors showed that injection of zebrafish embryos with this MO prevented gel mobility shift of radiolabeled nucleotides containing a heat shock response element when incubated with embryo lysates. Here, we examined HSF1 expression in embryos by Western blotting. Figure 4A shows a representative Western blot of protein obtained from non-transfected HeLa cells and cells transfected with plasmid coding for expression of an enhanced green fluorescent protein (EGFP) alone or a zebrafish HSF1-EGFP fusion protein. A band is detected in all samples at the predicted molecular weight of human HSF1 (huHSF1), and another is seen at the predicted molecular weight of the zebrafish HSF1-EGFP fusion protein in samples obtained from cells transfected with plasmid coding for the fusion protein, but not for EGFP alone. Figure 4B is a representative Western blot of nuclear protein in 48 hpf embryos following injection with a 0.25 mM solution of either a control or α-HSF1 MO. A single band of approximately 150 kDa was strongly detected in samples obtained from embryos injected with the control MO, but only weakly detected in embryos injected with the α-HSF1 MO. The position of this band relative to molecular weight markers (not shown) is consistent with the predicted molecular weight of activated HSF1 trimers (see [25] for review). We also examined effects of MO injection on the expression of a fluorescent reporter plasmid containing the zebrafish HSF1 promoter. Fluorescent cells were observed in 95% of embryos co-injected with the reporter construct and a control MO, but only 41% of embryos co-injected with the α-HSF1 MO (Fig. 4C). Finally, we examined effects of α-HSF1 MO injection on expression of heat shock proteins in embryos responding to heat shock (Fig. 4D). As expected, non-injected (WT) embryos showed an increase in Hsp70 and Hsp27 expression after heat shock. This upregulation was also observed in embryos injected with a concentration of α-HSF1 MO below the threshold needed to reduce HSF1 expression in Western blot assays (0.15 mM, data not shown). However, when embryos were injected with effective (0.3 mM) and excess (0.5 mM) α-HSF1 MO, heat shock-induced upregulation of Hsp27, although not Hsp70, was partially inhibited.

### Knockdown of HSF1 increases apoptosis in zebrafish brain and eye in response to HR

**Figure 5:**
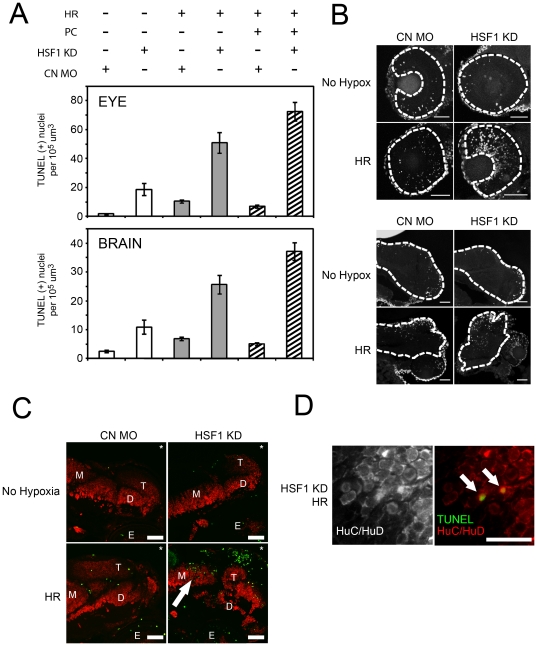
Effects of HSF1 knockdown on apoptosis. A: Quantification of TUNEL positive nuclei in the eye and brain of 58 hpf embryos, showing effects of HSF1 knockdown (compare HSF1 KD versus CN MO), in embryos incubated under control conditions (white bars), after hypoxia/reperfusion only (gray bars), and after heat shock preconditioning followed by hypoxia/reperfusion (striped bars). Error bars are the SEM. B: TUNEL positive cells in representative images of eye (upper panels) and brain (lower panels) regions after hypoxia. Dashed lines indicate the region in which nuclei were counted. Scale bars are 50 µm. C: Analysis of brain morphology and apoptosis after HSF1 knockdown. Representative images showing HuC/HuD immunostaining of neurons (red) and TUNEL positive nuclei (green) in brain sections obtained from embryos injected with control (CN) or HSF1 antisense (HSF1 KD) MO and incubated in normoxic medium (No Hypoxia) or exposed to hypoxia and reperfusion (HR). Asterisks mark the dorsal, anterior aspect of the embryos. Labels are telencephalon (T), midbrain (M), diencephalon (D), and eye (E). Scale bars are 50 µm. D: Representative higher magnification images showing localization of markers for neurons and apoptotic cells in cryosectioned brain. Scale bars are 10 µm.

To examine the role of HSF1 in brain and eye tissues, we injected embryos with either the control or the α-HSF1 MO. We then quantified the number of apoptotic cells in tissue sections obtained from embryos incubated under control conditions, after sublethal HR alone, and after heat shock preconditioning followed by sublethal HR (Fig. 5A). Images shown in Figure 5B are representative z-series projections containing the number of apoptotic nuclei closest to the corresponding averages shown in Figure 5A. In the absence of injury, embryos injected with control-MO had an average of 1.8+/−0.3 (eye) and 2.5+/−0.4 (brain) TUNEL positive nuclei per unit volume, compared to 18.7+/−4.0 (eye) and 10.9+/−2.4 (brain) in HSF1 knockdown embryos (white bars, Fig. 5A). Although these differences are large and statistically significant (p<0.001), note that the absolute numbers of TUNEL positive nuclei in these embryos are small relative to the values seen in embryos exposed to HR (below).

Next, we examined effects of HSF1 knockdown in embryos after a sublethal (100 min) exposure to HR. Embryos injected with the control MO had an average of 10.4+/−1.2 (eye) and 6.8+/−0.6 (brain) TUNEL positive nuclei per unit volume after HR, compared to 50.9+/−7.1 (eye) and 25.7+/−3.2 (brain) nuclei in those injected with the α-HSF1 MO (gray bars, Fig. 5A). Thus, knockdown of HSF1 expression significantly (p<.001) increased the number of TUNEL positive nuclei about 5-fold in the eye and 4-fold in the brain compared to embryos injected with the control MO.

Finally, we examined the effects of α-HSF1 and control MO injection on embryos subjected to heat shock preconditioning prior to HR (striped bars, Fig. 5A). The number of TUNEL-positive nuclei per unit volume in control MO injected embryos subjected to heat shock preconditioned before HR was 7.0+/−1.1 (eye) and 5.1+/−0.5 nuclei (brain). Therefore, for embryos injected with the control MO and subjected to non-lethal HR, preconditioning had a small but measureable protective effect, reducing the average number of apoptotic cells per unit volume from 10.4 to 7.0 in the eye and from 6.8 to 5.1 in the brain. In contrast, no protective effect of heat shock preconditioning was seen in embryos injected with the α-HSF1 MO. For these embryos, the number of TUNEL positive nuclei observed after sublethal HR was 72.6+/−6.4 (eye) and 37.1+/−3.1 (brain). This corresponds to an approximately 10.9-fold and 7-fold increase in apoptotic cell death compared to embryos subjected to the same conditions but injected with the control MO. These differences are statistically significant (p<0.001).

In summary, for both brain and eye tissues, levels of apoptosis were greater under all conditions examined in embryos injected with α-HSF1 MO, as compared to embryos injected with a control-MO (Fig. 5).

As there are many cell-types in the brain, we also addressed whether cells undergoing apoptosis in the above studies of brain tissues were neurons. To accomplish this, embryos were sectioned after treatment and processed for TUNEL staining and immunostaining with an anti-HuC/HuD antibody specific for differentiated neuronal cells. Under control conditions, overall development of neuronal cells in the brain, as assessed by the distribution and extent of anti-HuC/HuD staining, did not appear to be affected by HSF1 knockdown (Fig. 5C, compare CN MO to HSF1 KD in panels labeled “No Hypoxia”). After hypoxia, embryos injected with both control and α-HSF1 MO displayed TUNEL positive nuclei in brain regions that consisted almost exclusively of HuC/HuD positive neurons under control conditions (compare Fig. 5C, arrow and Fig. 5C, panels labeled “No Hypoxia”). In addition, upon higher magnification, TUNEL positive nuclei were seen in cells stained with the anti-HuC/HuD antibody (Fig. 5D).

### Effects of HSF1 knockdown on HSP expression after preconditioning or HR treatment

**Figure 6:**
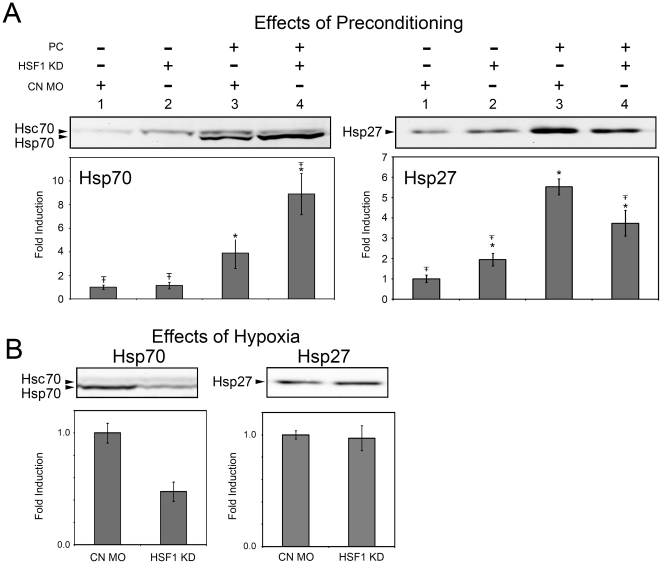
Alterations in heat shock protein expression following HSF1 knockdown. A: Representative Western blots, and quantitative analysis of Hsp70 and Hsp27 expression. Expression was analyzed in 48 hpf embryos injected with control (CN MO) or HSF1 antisense (HSF1 KD) morpholinos incubated at control temperatures or subjected to heat shock preconditioning (PC). Values represent average data from four independent experiments. Error bars are the SD, symbols indicate a significant difference from CN-MO injected embryos that were not preconditioned (*) and from CN-MO injected embryos that were preconditioned (Ŧ–). B: Representative Western blots and quantitative analysis of Hsp27 and Hsp70 expression after a 100 min hypoxia treatment. Embryos were injected with HSF1 antisense MO (HSF1 KD) or a control MO (CN). Bars represent data collected from three independent experiments. Error bars are the SEM.

To examine the relationship between HSF1 and heat shock protein expression, we analyzed the effects of HSF1 knockdown on the expression of Hsp27 and Hsp70 after preconditioning (Fig. 6A) and HR (Fig. 6B). Under control conditions, no Hsp70 was detected on these blots (Fig. 6A lanes 1,2) consistent with a previous study showing expression of Hsp70 only in the embryonic zebrafish lens [26]. Heat shock preconditioning produced a robust increase in Hsp27 and a modest increase in Hsp70 expression in embryos injected with control MO (compare lanes 1 and 3 in Fig. 6A). Injection of the α-HSF1 MO alone resulted in a small, but statistically significant, increase in expression of Hsp27, but not Hsp70 (compare lanes 1 and 2). Surprisingly, in heat shock preconditioned embryos, Hsp70 was expressed at significantly higher levels in HSF1 knockdown embryos than in embryos injected with control-MO (Fig. 6A compare Hsp70 lanes 3 and 4, p<.001). In contrast, injection of the α-HSF1 MO significantly decreased Hsp27 expression resulting from heat shock preconditioning (Fig. 6A compare Hsp27 lanes 3 and 4, p<.001). These data show that Hsp70 and Hsp27 have unique patterns of regulation following heat shock and that heat shock-induced upregulation of HSPs persists despite a significant reduction in HSF1 expression.

Finally, we examined the expression of Hsp27 and Hsp70 in MO injected embryos after 100 min of hypoxia and 8 hours of reperfusion (Fig. 6B). Although expression of both proteins was elevated as compared to embryos incubated in normoxic media (data not shown), embryos injected with the α-HSF1 MO exhibited decreased Hsp70 levels after hypoxia. However, Hsp70 expression was detectable by Western blotting in samples obtained from HR treated embryos despite injection of α-HSF1 MO (Fig. 6B), whereas Hsp70 was not detectable in samples obtained from control embryos (Fig. 6A, lanes 1,2). In addition, HSF1 knockdown had no effect on Hsp27 expression levels in embryos after hypoxia treatment (Fig. 6B).

## Discussion

### The resistance of zebrafish embryonic brain and eye tissues to hypoxia/reperfusion injury requires HSF1

Ischemia/reperfusion injury (IR) plays an important role in a variety of clinical pathologies including stroke and retinopathy [1]. The initial response of cells to low oxygen is largely mediated through actions of the hypoxia inducible factor (HIF) family of proteins (reviewed in [27]). However, abundant data support the view that the activation of stress inducible factors such as heat shock factor 1 (HSF1), and the subsequent expression of cytoprotective heat shock proteins, have critical roles in cell survival during tissue reoxygenation. For example, HSF1 is expressed in cells of the mammalian brain and eye, and activation of HSF1 in these tissues by IR and other stressors has been previously demonstrated [14], [28], [29], [30]. Additionally, experimental manipulations that elevate heat shock protein expression in the brain and eye activate HSF1 and confer resistance to IR [16], [31], [32].

The present study directly examined the protective role of HSF1 in the embryonic zebrafish eye and brain using a gene knockdown approach. Relatively few studies have examined the expression or function of heat shock transcription factors in teleosts, but available data support the view that zebrafish HSF1 is functionally equivalent to its mammalian orthologs. For example, zebrafish HSF1 shares 64% identity to human HSF1 and is 91% identical to the human protein within its DNA binding domain [18]. Additionally, data from the present study and previous work have demonstrated the ability of HSF1 knockdown to abrogate heat-induced expression of heat shock proteins [24]. Similarly, HSF1 null mice are unable to upregulate heat shock proteins following heat stress [20], [33].

Our results demonstrate that suppression of HSF1 expression results in a dramatic increase in apoptotic cell death in brain and eye tissues of zebrafish embryos subjected to hypoxia/reperfusion injury. Although the design of our studies did not allow us to identify specific neuronal cell types or brain regions showing HSF1 dependence, it seems clear that this cell death could have devastating effects on brain and eye function, especially if the data obtained from thin sections is extrapolated to the entirety of the brain and eye. Our results are consistent with previous studies demonstrating a protective role for HSF1 in neurons and other cell types, but contrast with some studies demonstrating the lack of HSF1 expression or activity in specific neurons and the aging brain. Specifically, others report that some neurons express little or no HSF1 [34], [35], and the ability of isolated neuronal cells to activate HSF1 and synthesize heat shock proteins in response to stressful stimuli declines during differentiation [36], [37], a phenomenon also observed in the aging brain [38], [39]. The zebrafish embryo is a highly dynamic developmental system, and brain and eye tissues of tested animals were not fully differentiated at the time of our experiments. Therefore, the observed increase in HR-induced apoptosis in HSF1 knockdown embryos may be in a cell population that normally contains higher levels of HSF1 than their fully differentiated counterparts. However, brains of embryos at the stage of development used in our experiments express markers for differentiated neurons ([40], [41] and Fig. 5). In addition, retinal cell differentiation is evident in the zebrafish embryo by 48 hpf [42]. Therefore, our results demonstrating an essential role for HSF1 in cell survival after IR are likely relevant to differentiated brain and eye tissues.

### The role of HSF1 in resistance to IR may be independent of Hsp27 and Hsp70 expression

Expression of heat shock proteins, such as Hsp27 and Hsp70, has been shown to protect cells against IR in a variety of model systems. However, results of the present study show that both Hsp70 and Hsp27 are expressed in HSF1 knockdown embryos, yet these embryos none-the-less display elevated levels of apoptotic cell death after HR compared to control MO injected animals. These data suggest that there are essential functions for HSF1 other than the promotion of heat shock protein expression in the brain and eye, and are consistent with recent studies showing increasingly complex roles for HSF1 in development and tissue homeostasis. For example, *Drosophila* HSF is capable of binding to nearly 3% of genes [43] and recent reports indicate that vertebrate HSF1 also binds to and activates numerous genes outside the canonical stress response pathway [44]. Additionally, in HSF1-null mice, cardiac [45] and renal cells [46] redox homeostasis is impaired through still poorly defined mechanisms. Inability to properly regulate redox states could make highly metabolically active tissues, such as found in the brain, unusually vulnerable to the oxidative damage of IR. HSF1 null mice also have altered glucose uptake and metabolism [47]. Animals lacking HSF1 may be unable to undergo critical alterations in glucose metabolism during hypoxic challenge. Therefore, it is unsurprising that the lack of HSF1 has deleterious effects in zebrafish embryos despite the presence of Hsp27 and Hsp70.

### Hsp27 and Hsp70 expression may be induced by IR through a mechanism other than HSF1 activation

Our results are consistent with previous studies showing the ability of HSF1 to protect cells against IR, but are novel in showing that Hsp70 and Hsp27 expression persists after HR despite substantial reduction in HSF1 expression. It is possible that these results reflect limitations of our methods. For example, although we demonstrate effective knockdown of HSF1 by Western blot, morpholino microinjection, unlike gene deletion methods, does not completely eliminate HSF1 expression. The quantity of morpholino injected into embryos in the present study was sufficient to inhibit heat shock-induced upregulation of Hsp27 (Fig. 4). However, the expression of Hsp70 was not similarly inhibited. We conclude that HSP expression is regulated independent of HSF1 in embryos subjected to HR, but cannot rule out the possibility that upregulation of Hsp70 and perhaps other HSPs simply requires less activated HSF1 than upregulation of Hsp27. Our data showing HSF1 independent expression of Hsp70 also conflict with the results of at least one previous study showing HSF1 dependent expression of Hsp70 in heat shocked zebrafish embryos [24]. However, recovery times used in our studies were longer than those used in this previous study, possibly allowing upregulation of Hsp27 though slower acting and HSF1 independent mechanisms. We note that our results are also consistent with recent results showing that the heat shock response was unaltered in Xenopus tadpoles after RNAi-based inhibition of HSF1 expression [48].

How might HR regulate heat shock protein expression independent of HSF1? Although stress-dependent heat shock protein expression is primarily regulated by HSF1, alternative mechanisms can clearly do so. For example, HIF-1 has been shown to directly induce expression of both Hsp27 [49] and Hsp70 [50]. HIF-1 also regulates expression of members of the HSF family [51]. Since zebrafish, like mammals, have multiple HSFs, it is possible that upregulation of, or compensation by, other HSFs may be responsible for HSF1-independent heat shock protein upregulation observed in our system.

Overall, our results reveal that HSF1 plays a critical role in preventing apoptotic cell death in brain and eye tissues after HR injury. However, although HSF1 is required for expression of Hsp27 induced by heat shock, reduced expression of HSF1 had little effect on Hsp70 expression and no detectable effect on the enhanced expression of Hsp27 in zebrafish embryos after HR. Therefore, elevated heat shock protein expression after HR appears to be independent of HSF1 and is likely necessary, but not sufficient to prevent cell death in the absence of functional HSF1. These results suggest alternative roles for HSF1 in cellular resistance to IR injury.

## Materials and Methods

### Zebrafish husbandry and heat shock preconditioning

Adult zebrafish, (*Danio rerio*, Tuebingen strain), were cared for according to previously established protocols [52]. Embryos were collected and reared in filtered fish water (ddH2O containing 0.125 g/L of NaHCO3, 0.18 g/L of Instant ocean, 1 mg/L methylene blue and 100 µg/ml ampicillin). Embryos injected with morpholino or plasmid solutions were reared in embryo media (13.7 mM NaCl, 0.54 mM KCl, 0.025 mM Na_2_HPO_4_, 0.044 mM KH_2_PO_4_, 1.30 mM CaCl_2_, 1 mM MgSO_4_ × 7H_2_O, 4.17 mM NaHCO_3_, pH 7.2) for 6 hours, then transferred to fish water. All embryos were screened for appropriate stage development at 30 hours post fertilization and manually dechorionated. Heat shock preconditioning was accomplished by placing dechorionated 34 hour post fertilization embryos in 6 cm petri dishes containing 9 mL of fish water. After sealing, petri dishes were immersed for 90 min in a 37°C circulating water bath (IsoTemp 2150, Fisher Scientific, Pittsburgh, PA, USA). Protocols for the use of animals in these experiments were approved by the Washington State University Animal Care and Use Committee and were in accord with National Institute of Health standards established by the Guidelines for the Care and Use of Experimental Animals.

### Hypoxia/Reperfusion treatment

Filtered fish water was boiled to remove dissolved gasses. After addition of 0.2 mg/ml sodium sulfite, the water was cooled on ice under nitrogen gas to 28.5°C. Oxygen levels were measured at the start and end of experiments with a YSI DO200 O_2_ meter. All dissolved oxygen readings for these experiments were between 0.08 and 0.12 ppm O_2_ at 28.5°C. Embryos were added to chambers (250 ml flasks with airtight lids) containing deoxygenated water for the indicated period. Embryos were then placed in 24 well plates containing prewarmed normoxic fish water at a density of 1 embryo/well to recover until harvest for analysis.

### TUNEL analysis

Fluorometric terminal deoxynucleotidyl transferase dUTP nick end labeling (TUNEL) was accomplished with modified protocols using the Promega DeadEnd™ Fluorometric TUNEL system. Briefly, groups of greater than 20 embryos were fixed in 4% paraformaldehyde for 1 hour at room temperature. Embryos were dehydrated in an ethanol series and stored at −20°C for at least 24 hours. After rehydration, embryos were permeabilized in lysis solution (20 mM Tris, 150 mM NaCl, 1 mM EDTA, 1 mM EGTA, 0.5% Triton X-100, pH 7.3) for 3 hours at 4°C and treated with 5 µg/mL proteinase K for 5 min at room temperature. Embryos were post-fixed in 4% paraformaldehyde for 30 min at room temperature then incubated for 10 min in equilibration buffer. Equilibration solution was removed and 50 µL of the standard TUNEL reaction solution was added. Embryos were stored in reaction solution overnight at room temperature then incubated at 37°C for one hour. The reaction was stopped by addition of 1 mL of 2× SSC buffer. Embryos were counterstained for total nuclei in 5 µg/mL of Hoechst in PBS for 4 hours at 4°C, then embedded in 100% OCT compound and frozen in liquid nitrogen. Longitudinal sections were cut at a thickness of 15 µm and mounted in PVA-glycerol mounting medium containing DABCO as an anti-fading agent. For quantitative TUNEL analyses shown in Figures 3 and 5, heads of 15 embryos in each test group were embedded, sectioned entirely and all obtained sections mounted on chrome-gelatin coated coverslips. A through-focus series (z-series) of images was obtained of all sections containing identifiable brain or eye tissues at 2 µm intervals using a confocal microscope (Zeiss LSM 510 META, Carl Zeiss Inc., Thornwood, NY, USA) equipped with a 0.8 NA 25× glycerol immersion lens. Pinholes were adjusted to one Airy disc unit for each fluorophore and images acquired using a pixel dimension of 0.75 microns. Gain and black levels settings were optimized for each image to avoid over- and under-saturation. We estimate that our methods generated an average of 10 useable sections per embryo. However, we produced images of fewer sections due to distortion and losses during staining and mounting procedures. In total, images were produced for a minimum of 68 and a maximum of 130 sections for each test condition (data not shown). To quantify apoptotic nuclei, thirty z-series representing each test condition were randomly selected for further analysis and viewed using ImageJ (Image J version 1.37, Wayne Rasband, National Institute of Health, Bethesda, MD). TUNEL-positive nuclei appearing in individual image planes were marked and counted with a mouse-driven cursor. Nuclei appearing in more than one image plane were counted only once. Values shown in figures are the number of counted nuclei per unit volume, calculated by multiplying the 2-dimensional area of the region of tissue under examination by the number of optical sections and the section thickness. Separate analyses were conducted for eye and brain tissues, but no attempt was made to track sections from individual embryos or identify specific sub-regions of these tissues. Counts of sections analyzed, total TUNEL (+) nuclei observed, and average area of the sections in each condition are presented in Tables S1 and S2. Images shown in Figures 3B and 5B were created using a “brightest pixel” projection feature of ImageJ and contrast adjusted using Adobe Photoshop CS4.

### SDS-PAGE and Western Blotting

SDS-PAGE and Western blotting was carried out essentially as described [53]. For assays of heat shock protein expression, embryos were chilled on ice and yolks removed. Samples were then solubilized in 2× SDS-PAGE sample buffer, sonicated briefly and boiled for 5 min. For analysis of HSF1 protein expression, 48 hpf embryos were heat shocked for 45 min at 37°C to cause translocation of HSF1 into the nucleus. Embryos were chilled on ice and immersed for 10 min at room temperature in a fractionation buffer (10 mM HEPES (pH 7.9), 10 mM KCl, 0.1 mM EDTA, 0.4% IGEPAL, 1∶1000 protease inhibitor cocktail (Sigma Aldrich P8340)). Embryos were homogenized using a Dounce homogenizer, then centrifuged for 5 min. Pellets, containing nuclei, were resuspended in solubilization buffer (20 mM HEPES (pH 7.9), 0.4 M NaCl, 1 mM EDTA, 10% glycerol, 1∶500 protease inhibitor cocktail) for 4 hours at 4°C and then mixed with an equal volume of 2× SDS-PAGE sample buffer. Protein concentrations were determined using a BioRad D_C_ Protein Assay. 20 µg of protein was loaded per well and separated with a 4% stacking and a 12% (for Hsp27) or 10% (for Hsp70 and HSF1) running gel. Proteins were transferred to nitrocellulose membranes and stained with Ponceau S to confirm equal protein loading. Immunoblotting was accomplished using an α-Hsp27 antiserum [53], an α-Hsp70 antibody (SPA-812, Stressgen) or an α-HSF1 antibody (SPA-901, Stressgen). For analysis of Hsp70 expression, SDS PAGE was performed for times sufficient to separate Hsc70 from Hsp70, as described by Evans et al. [24]. Chemiluminescent detection and integrated optical density (IOD) measurements were performed as described [54]. Approximate molecular weights of observed bands were 150 kDa (HSF1), 22 kDa (Hsp27), and 70 kDa (Hsc70 and Hsp70). Under the conditions used for these assays, Hsp70 migrated slightly faster than Hsc70 and could be resolved as a distinct lower band in resulting anti-Hsp70 Western blots (see Figures 2, 4 and 6). Each experiment was performed at least three times. Integrated optical density of bands was quantified using ImageJ as described previously [54].

### Molecular cloning and exogenous expression of HSF1 plasmid constructs

A full-length cDNA encoding zebrafish HSF1 (IMAGE clone 8754667, Thermo Scientific) was amplified using forward and reverse primers (5′-CTAATTACCTGCAGAAATCATCACTAAACTCTCC-3′ and 5′-GGTACCGAAGCTGGTACTTTTTGACTGC-3′, respectively) and Platinum Pfx DNA polymerase (Invitrogen). The resulting PCR product was digested with Pst-I and cloned into the Pst-I restriction site of plasmid pEGFP-C1 (Clontech) using standard cloning methods. HeLa cells were transfected with pEGFP-C1-HSF1 using Effectene (Qiagen) according to the manufacturer's protocol. Cells were seeded at a density of 5×10^4^ cells/well in 24-well plates and incubated for 24 hrs in a 5% CO_2_ incubator. Cells were then washed with MEM, and DNA-enhancer mixture (400 ng DNA, 1.6 µL enhancer, 5 µL Effectene, 56.4 µL EC buffer, 750 µL MEM) was added. After overnight incubation, cells were washed in PBS and collected in 2× SDS sample buffer. To create the zfHSF1 promoter reporter construct, pEGFP-C1 was modified by the addition of a Not-I site by digestion with the enzyme Ase-I and ligation with oligonucleotides 5′-TAATGCGGCCGC-3′ and 5′-ATGCGGCCGCAT-3′. The zebrafish HSF1 promoter was amplified from zebrafish genomic DNA using forward and reverse primers 5′-CGCGGCCGCCATCTTAGCCGTGAGAAG-3′ and 5′-GACCGGTAGCGTCCAGAGTTTGGTCAGAAACG-3′ and Platinum Pfx DNA polymerase (Invitrogen). The resulting PCR product was digested with Not-I and Age-I and cloned into the same restriction sites of the modified pEGFP-C1 using standard cloning methods. All constructs were sequenced using fluorescent dye-terminator biochemistry (Big Dye, Applied Biosystems, Foster City, CA) and analyzed using an ABI Prism 377 automated gel reader (Perkin-Elmer Corp.). Expression of the resulting coding sequence results in production of a fusion protein containing the first 28 amino acids of zebrafish HSF1 linked to EGFP at the C-terminal end by the amino acid sequence PVAT.

### Immunolocalization

Embryos were chilled on ice and fixed in 4% paraformaldehyde for 1 hour at room temperature. Embryos were then embedded in 100% OCT and frozen in liquid nitrogen. Heads were sectioned at a thickness of 15 µm and sections placed on chrome-gelatin subbed coverslips. Sections were permeabilized in Tris-lysis solution for 5 min, then blocked for 1 hour in PBS containing 0.1% Tween-20 (PBS-Tw) and 10% calf serum. Sections were incubated in PBS-Tw with primary antibodies (SPA-812 for Hsp70 (1∶250), mouse anti-HuC/HuD (Invitrogen, 1∶50), and rabbit anti-zebrafish Hsp27 antiserum (1∶250)) overnight at 4°C in a humid chamber. FITC-labeled (for HSPs) or Texas-red labeled (for anti-HuC/HuD) secondary antibodies were used at a concentration of 1∶500 for 3 hours at room temperature. Samples were mounted using PVA-glycerol mounting medium containing DABCO. Images were obtained with a Zeiss LSM 510 META confocal microscope using a 63×, 1.4 NA oil immersion lens and a pixel dimension of 0.14 microns.

### Microinjection

Morpholino oligonucleotides (MO) were purchased from Genetools LLC (Corvallis, OR) and dissolved in injection buffer (0.4 mM MgSO_4_, 0.6 mM CaCl_2_, 0.7 mM KCl, 58 mM NaCl, 25 mM HEPES pH 7.1). The sequence for the HSF1 antisense MO was described previously [24], [33]. To control for toxicity associated with MO injection, all results obtained using the HSF1 antisense MO were compared to those obtained using a non-specific control MO described previously [53]. Needles for injection were pulled from borosilicate capillary tubes (1.00×0.78 mm×10 cm, Sutter Instruments Co., Novato, CA, USA) using a Sutter Instruments pipette puller (Model P-87). Approximately 1.8 nL of MO were injected into yolks of embryos prior to the two cell stage using methods outlined by Liu and coworkers [55]. The concentration of MO needed to prevent HSF1 expression was determined by injecting a series of different MO concentrations and evaluating HSF1 expression by Western blotting (data not shown). The minimal concentration at which no further reduction in HSF1 expression could be generated (0.25 mM) was used for all subsequent experiments. For plasmid injection, HSF1-EGFP reporter constructs were injected at 30 ng/µL with a final injection volume of 1.8 nL.

### Statistical Analyses

Averages of values for single comparison analyses are displayed with the calculated standard error of the mean. Normality was assessed by qualitative analysis of histogram distributions. Comparisons between two samples were performed using Student's *t* test. For multiple comparisons, averages are displayed with calculated standard deviation. Statistical comparisons for these experiments were performed using two-way ANOVA followed by Tukey's W procedure post-hoc test. Calculated p-values of less than or equal to 0.05 were considered statistically significant.

## Supporting Information

Table S1 **TUNEL data obtained following heat shock preconditioning and/or hypoxia and reperfusion in uninjected embryos.** Data were obtained from TUNEL (+) nuclei counts of zebrafish head sections. “Number of sections” is the total number of random sections analyzed. “Total nuclei” are the total number of TUNEL (+) nuclei counted in all sections.
(DOCX)

Table S2 **TUNEL data obtained following morpholino (MO) microinjection, heat shock preconditioning and/or hypoxia and reperfusion in zebrafish embryos.** Data were obtained from counts of TUNEL (+) nuclei in embryos subjected to injection with control (CN) or HSF1 antisense (HSF1 KD) MO, followed by preconditioning (PC), and/or hypoxia/reperfusion (HR). “Number of sections” is the total number of random sections analyzed. “Total nuclei” is the total number of TUNEL (+) nuclei counted in all sections.
(DOCX)
