# Supplementary material for: HSF1 Is Essential for the Resistance of Zebrafish Eye and Brain Tissues to Hypoxia/Reperfusion Injury
Source: PLoS One. 2011 Jul 21;6(7):e22268. doi: 10.1371/journal.pone.0022268 (PMC3141033; doi:10.1371/journal.pone.0022268)
Supplement: Table S1 — TUNEL data obtained following heat shock preconditioning and/or hypoxia and reperfusion in uninjected embryos. Data were obtained from TUNEL (+) nuclei counts of zebrafish head sections. “Number of sections” is the total number of random sections analyzed. “Total nuclei” are the total number of TUNEL (+) nuclei counted in all sections. (DOCX) [file pone.0022268.s001.docx]

| EYE |  |  |  |
| --- | --- | --- | --- |
| Condition | Number of sections | Total nuclei | Average volume per section (μm^3^) |
| NoPC | 30 | 2643 | 372159 |
| PC | 30 | 759 | 343599 |
|  |  |  |  |
| BRAIN |  |  |  |
| Condition | Number of sections | Total nuclei | Average volume per section (μm^3^) |
| NoPC | 30 | 3843 | 821325 |
| PC | 30 | 1102 | 839133 |
