# Supplementary material for: HSF1 Is Essential for the Resistance of Zebrafish Eye and Brain Tissues to Hypoxia/Reperfusion Injury
Source: PLoS One. 2011 Jul 21;6(7):e22268. doi: 10.1371/journal.pone.0022268 (PMC3141033; doi:10.1371/journal.pone.0022268)
Supplement: Table S2 — TUNEL data obtained following morpholino (MO) microinjection, heat shock preconditioning and/or hypoxia and reperfusion in zebrafish embryos. Data were obtained from counts of TUNEL (+) nuclei in embryos subjected to injection with control (CN) or HSF1 antisense (HSF1 KD) MO, followed by preconditioning (PC), and/or hypoxia/reperfusion (HR). “Number of sections” is the total number of random sections analyzed. “Total nuclei” is the total number of TUNEL (+) nuclei counted in all sections. (DOCX) [file pone.0022268.s002.docx]

| EYE |  |  |  |
| --- | --- | --- | --- |
| Condition | Number of sections | Total nuclei | Average volume per section (μm^3^) |
| CN MO no hypox, no PC | 23 | 77 | 159079.9 |
| HSF1 KD no hypox, no PC | 18 | 540 | 152339 |
| CN MO hypox, no PC | 30 | 741 | 260914 |
| HSF1 KD hypox, no PC | 30 | 2108 | 178268 |
| CN MO hypox, PC | 30 | 574 | 281507 |
| HSF1 KD hypox, PC | 30 | 2741 | 128788 |
|  |  |  |  |
| BRAIN |  |  |  |
| Condition | Number of sections | Total nuclei | Average volume per section (μm^3^) |
| CN MO no hypox, no PC | 22 | 228 | 475967 |
| HSF1 KD no hypox, no PC | 17 | 776 | 490158 |
| CN MO hypox, no PC | 30 | 1108 | 561481 |
| HSF1 KD hypox, no PC | 30 | 3761 | 784964 |
| CN MO hypox, PC | 30 | 881 | 578627 |
| HSF1 KD hypox, PC | 30 | 4471 | 425215 |
